# Injectable functionalised Lycium barbarum polysaccharide/alginate hydrogel particles for the treatment of bone defects

**DOI:** 10.1016/j.mtbio.2025.101919

**Published:** 2025-05-28

**Authors:** Kai Xie, Zhengwei Liu, Yansong Wang, Lei Zhu, Jiazhao Yang, Yongxiang Wang, Mingming Zhang

**Affiliations:** a Department of Orthopedics, Northern Jiangsu People's Hospital, Clinical Teaching Hospital of Medical School, Nanjing University, Yangzhou, 225009, China; b Shenzhen Hospital (Futian) of Guangzhou University of Chinese Medicine, China; c Department of Orthopaedics, The First Affiliated Hospital of University of Science and Technology of China, Hefei, 230001, Anhui, China; d Department of Rheumatology, The First Affiliated Hospital of Anhui Medical University, Hefei, 230022, China

**Keywords:** Barbarum polysaccharides, Sodium alginate, Tissue engineering, Hydrogel, Bone defects

## Abstract

The treatment of bone defects presents a number of significant clinical challenges. In recent years, plant-derived hydrogels have demonstrated considerable potential as a means of intervening in bone defects. In this study, a alginate hydrogel (Alg) system containing the Chinese medicinal plant Lycium barbarum was prepared with the objective of promoting the repair of bone defects. The hydrogel system was composed of Lycium barbarum polysaccharide (LBP), sodium alginate, and Srcl_2_. The study demonstrated that the hydrogel system was injectable and capable of rapid cross-linking to form a gel, rendering it suitable for bone defects of varying shapes. In vitro studies have shown that hydrogels exhibit good biocompatibility and have also been shown to promote bone and vascular regeneration. In animal experiments, the hydrogel system was observed to elicit bone-enhancing and angiogenic effects through the sustained release of active ingredients within the bone defect region. These findings suggest that the Sr-LBP/Alg hydrogel system may offer a potential avenue for bone tissue engineering and regenerative medicine.

## Graphical abstract

**Figure:**
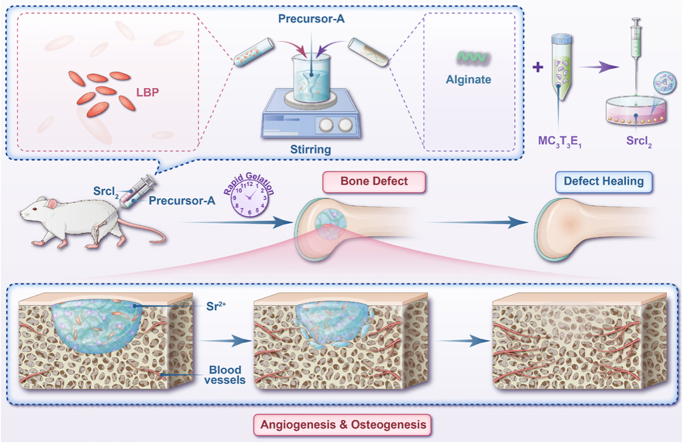

## 1 Introduction

The incidence of traumatic bone defects has been increasing in recent years [1], and autograft and allograft represent two of the more frequently utilised options for the treatment of bone defects [[2], [3], [4], [5]]. However, the availability of autologous bone for grafting is constrained, and the potential for complications exists. Consequently, the search for an alternative therapeutic strategy that promotes bone regeneration with superior bone-promoting properties, minimal invasiveness, slow-release and targeted delivery is of great importance. The field of bone tissue engineering has reached a considerable degree of maturation, and biologic factors and drugs also play a significant role in the repair of bone defects [[6], [7], [8], [9], [10]].

Lycium barbarum is a traditional Chinese medicine that has gained popularity in Western countries as an anti-aging product [11]. LBP is a polysaccharide compound comprising glucose, arabinose, xylose, galactose, mannose, rhamnose, and other monosaccharides [12,13]. It has been documented in the Compendium of Materia Medica that it strengthens muscles and bones. Zhang et al. [14] reported that LBP promoted the proliferation of osteoblastic MC3T3-E1 cells. Sun et al. [15] observed that LBP can enhance bone mass and strength, as well as the proliferation, differentiation, and ossification of osteoblasts. Moreover, the principal active substances of LBP that elicit the aforementioned effects and their potential mechanisms and targets were investigated. In conclusion, LBP demonstrated significant increases in bone mass and bone strength, as well as facilitating the proliferation, differentiation and ossification of osteoblasts and has the potential for application in the promotion of bone regeneration. Strontium (Sr) is a micronutrient found in bone that regulates the stimulation of osteoblast differentiation through the activation of the FAK/RhoA signalling pathway. This process promotes the expression of collagen type 1 (COL1), nt-associated transcription factor 2 (RUNX-2), osteocalcin (cyanate), and alkaline phosphatase (ALP), as well as matrix mineralization [16]. Furthermore, it has been observed that Sr also possesses the capacity to stimulate vascular regeneration and to inhibit inflammation [17]. In addition, a key role of Sr2+ is its excellent ionic cross-linker properties, which enable it to be used in the formation of alginate hydrogels [18]. Therefore, Sr ions renders strontium bone tissue engineering materials a prevalent choice for bone repair.

The effective control of the slow release of drugs remains a significant challenge in the field of bone defect repair. Local slow-release drug delivery systems promote bone repair by extending the drug release cycle [19,20]. Alginate hydrogel holds considerable potential as a drug delivery vehicle in tissue regeneration owing to its biocompatibility [21,22], which can imitate the natural extracellular matrix (ECM), thereby creating an optimal environment for cells to flourish [23,24]. Polysaccharide hydrogels are widely used in tissue engineering and regenerative medicine for their ability to enhance cell adhesion and proliferation in a manner similar to the natural cellular environment [25]. Injectable hydrogels particles provide a choice for minimally invasive treatments [26] and are suitable for complex shaped bone defects without inhibiting bone growth [27]. Based on the inherent drug delivery properties of hydrogels, they are excellent systems to load and control the release of LBP/Sr^2+^.

**Fig. 1:**
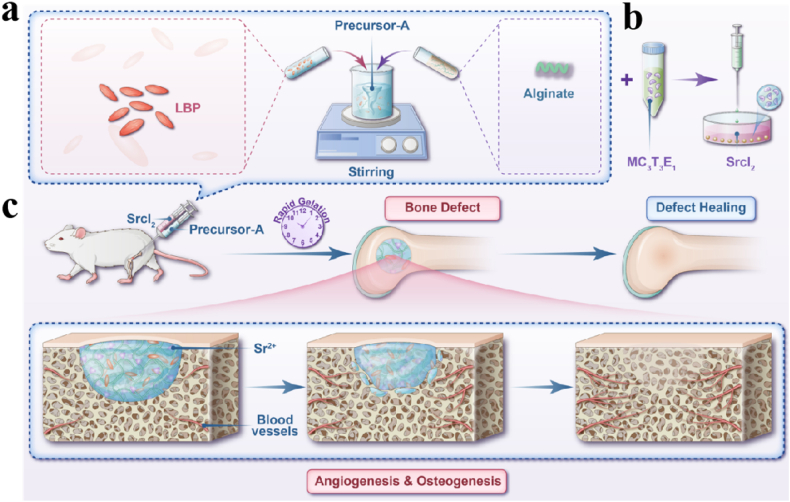
Schematic representation of an injectable, functionalized LBP/Alg composite hydrogel microsphere for the treatment of bone defects. a) Preparation process of injectable hydrogels. b) Schematic representation of the hydrogel-encapsulated cells. c) Schematic representation of Sr-LBP/Alg hydrogel to treat bone defects.

In this study, we designed injectable Sr-LBP/Alg hydrogels. The LBP and Alg were combined in varying proportions (1/3, 2/3, and 3/1) and introduced to one of the channels of the dual-channel syringe pump. The other channel was filled with a 5 % SrCl_2_ solution. When the syringe pump was operational, the two solutions were able to converge at the end of the syringe pump, forming an instant gel (Fig. 1). The effects of various LBP/Alg ratios on the morphology, structure, water absorption, and mechanical properties of hydrogels were assessed. The effects of Sr-LBP/Alg hydrogels on the multiplication and diversification of mouse embryo osteoblast precursor cells (MC3T3-E1) were determined through a series of evaluations. The ability of Sr-LBP/Alg hydrogels to promote bone defect repair was verified further using a rat femoral defect model. The results showed that the Sr-LBP/Alg hydrogel was able to promote bone defect repair. Therefore, we believe that Sr-LBP/Alg hydrogel has potential application in bone tissue engineering.

## 2 Experimental section

### 2.1 Materials

Sodium alginate (CP, G/M = 1 : 1, viscosity ( 200 ± 20 mPa s)) and SrCl2 (158.53 MW) were purchased from Mackin (China). Lycium barbarum polysaccharide (AP, Mw 22–25 kD, Purity ≥90 %) was purchased from Shengqing (China). LBP is composed of a variety of monosaccharides, including glucose, arabinose, xylose, galactose, mannose, and rhamnose. Of these, glucose and arabinose were present in concentrations exceeding 80 %. The structural information of these monosaccharides is presented in Supporting Information.

### 2.2 Preparation of the hydrogel

LBP at a concentration of 10 % (w/v) and Alg at a concentration of 3 % (w/v) were prepared with deionised water, and then Alg and LBP were mixed in the proportions of 1/3, 2/3 and 3/1, respectively, and stirred for 12 h at room temperature (25 °C) on an electric magnetic stirrer at 500 r/min. The LBP/Alg mixture was then dropped into 5 % SrCl_2_ solution to form a gel under the propulsion of a syringe pump. The hydrogel was collected and subjected to three washes with distilled water, with the objective of removing the insufficiently cross-linked polymer and residual SrCl_2_ from the material.

### 2.3 Characterisation of hydrogels

The prepared hydrogels were analysed in terms of their morphology and structure following a freeze-drying treatment, which was conducted using a vacuum freeze-dryer. Scanning electron microscopy (SEM) (APERO-S, America) was performed to survey the shape of the microspheres. Energy dispersive spectroscopy (EDS) (APERO-S, America) was used to analyse the variety of elements and their content in the micro-regions of materials.

### 2.4 Measurement of swelling ratio

After lyophilization of the hydrogel, the original mass of the sample was measured as W_0_. The hydrogels were immersed in 10 ml of Alpha medium at 37 °C and then weighed (W_t_) after removing the liquid on the surface of the hydrogel using a filter paper after 48 h. The samples were grouped on the basis of different ratios of Alg and LBP, and three samples from each group were taken for measurement.

$$\text{Swelling}\;\text{ratio}=W_{t}\;/\;W_{0}\text{.}$$

### 2.5 Hydrophilicity detection of hydrogels

The hydrophilicity of the surface of Alg and different ratios of LBP/Alg hydrogel materials was examined using a contact angle meter system (JC2000D1, China). The contact angles of the liquids on the surfaces of different hydrogel materials were measured by dropping 1 μL of ultrapure water on the surfaces of different hydrogel materials at room temperature.

### 2.6 Mechanical measurements

Rheoloical properties were carried out using rotation rheometer (MCR302, Austria) at 25 °C and 37 °C. Storage modulus (G′) and loss modulus (G″) tests were performed. In frequency scanning experiments, shear G′ and G″ were tested in a linear viscoelastic state with a frequency range of 0.1–10 Hz and a maximum strain γ of 0.2 %.

Compression tests were performed using a universal testing machine (AGS-X 50 N, Japan). The hydrogel was prepared in a cylindrical mold (5 mm in diameter and 5 mm in height). The compression rate was 5 mm/min. The compressive stress was calculated by dividing the load by the initial cross-sectional area. Each trial was repeated at least three times.

### 2.7 Mesh size evaluation and crosslinking density

On the basis of the Rubber Elasticity Theory (RET), the mean mesh size ξ is calculated as follows [28].

ξ=(G′NA/RT)‐(1/3)

In the equation, NA represents Avogadro's constant (6.022 × 10^23^), R denotes the molar gas constant (8.314 J/K mol), and T signifies temperature.

The crosslinking density of hydrogels represents another quantifiable parameter that can be determined through theoretical analysis. The crosslinking density (ne, mol/m^3^), defined as the volumetric concentration of elastically active network junctions, can be mathematically derived using the rubber elasticity theory (RET) according to the following relationship [29].

$$\eta_{e}=G_{e}\;/\;\text{RT}$$

where G_e_ is the plateau value of storage modulus measured by frequency sweep test.

### 2.8 Slow-release experiments

Alg and LBP were mixed at a ratio of 1/3 and added to 5 % (w/v) SrCl_2_ solution. The sample was placed in a 50 ml beaker containing PBS and then stirred slowly at 37 °C. The initial total concentration of LBP was C_0_, and the liquid was taken from the slow-release system at different times, measured with a UV spectrophotometer and converted to the concentration value of LBP C_t_.

$$\text{LBP}\;\text{release}\;\left(\%\right)=C_{t}\;/\;C_{0}\;*\;100$$

In order to detect the sustained release of LBP after implantation of the hydrogel system in vivo, we used P-phycoerythrin, which has a molecular weight comparable to that of LBP, instead of LBP to prepare the hydrogel system. The hydrogel was implanted subcutaneously in the lower limbs of SD rats, and as the P-phycoerythrin was able to fluoresce under specific wavelength excitation (488/575 nm), the release of LBP could be detected using animal imaging techniques at 0/5/10/15d.

### 2.9 In vitro degradation experiments

The initial weight (Wo) of the hydrogel after lyophilization was determined and then the hydrogel was immersed in PBS at room temperature. The residual weight (Wt) of the hydrogel after lyophilization was recorded at different time points. The residual mass of the hydrogel was calculated according to the formula.

$$\text{Remaining}\;\text{Mass}\;\left(\%\right)=\left(\text{Wt}\;/\;\text{Wo}\right)\;*\;100$$

### 2.10 Hydrogel encapsulation and cell culture

The MC3T3-E1 cultures were conducted using Alpha medium supplemented with 10 % fetal bovine serum (FBS, Gibco) and a penicillin-streptomycin solution (Biosharp). Third-generation MC3T3-E1 were digested and added to α-MEM medium to form a cell suspension, which was diluted with LBP/Alg in equivalent proportions. The substance was introduced into α-MEM with 5 % (w/v) SrCl_2_ via syringe. The obtained osteoblast hydrogel pellets were washed three times with α-MEM, and finally the osteoblast hydrogel spheres were transferred to 24-well plates and cultured. A description of the schematic diagram of the hydrogel-encapsulated osteoblasts is outlined in Fig. 1b.

### 2.11 Osteoblast proliferation

Osteoblast growth was measured by CCK-8(Beyotime Biotech Inc, Shanghai, China). Osteoblasts were embedded in LBP/Alg hydrogels with different LBP/Alg proportions (1/3, 2/3, and 3/1), respectively, and cultured. Osteoblast hydrogel spheres were lysed by adding 500 μL of a mixture of EDTA, Hepes and NaOH, pH 7.4, to each well. CCK-8 solution was incorporated to the wells and kept for 30 min in a 37 °C incubator. Absorbance was measured at 450 nm using an microplate reader (Multiskan, Thermo Scientific).

After 3 or 7 days, the hydrogel spheres were dissolved and the incubation was continued for 24 h. Cell viability/death staining was performed to detect viable MC3T3-E1 and non-viable MC3T3-E1 cells. Observation was performed with a Leica scanning microscope (CLSM, Leica, Germany).

### 2.12 Osteogenic and angiogenic differentiation

The osteogenic differentiation potential of Sr-LBP/Alg samples was assessed by evaluating alkaline phosphate (ALP) activity. MEC3T3-E1 cells were inoculated into well plates and subsequently co-cultured with the material. The normal medium was replaced with osteogenic induction medium Alpha-MEM (10 % FBS, 1 % PS, 10 mM β-glycerol phosphate, 50 μM l-ascorbic acid, 100 nm dexamethasone) after 1 day. Following a two-day interval, the medium was changed. Subsequent to a 14-day period of incubation, an alkaline phosphatase assay was conducted. The absorbance was determined by measuring the optical density at a wavelength of 520 nm. Alizarin red staining (ARS) was used to assess extracellular matrix mineralization. After 14 days of incubation, alizarin red staining was performed and the results were recorded by observation with a Lycra microscope. The ARS complex was solubilized using a cetylpyridinium chloride solution, and absorbance quantification was subsequently performed at 562 nm wavelength through spectrophotometric detection with a microplate reader.

The hydrogel was co-cultured with HUVECs for 2d. Add an appropriate amount of matrix gel to the 96-well plate to cover the well bottom and stand at room temperature for 30 min waiting for the matrix gel to solidify. The cell suspension prepared for HUVECs was added to a 96-well plate paved with Matrigel and added approximately 5000 cells per well. The cells undergo further cultivation for a period of 6–12 h. Obobserved under a light microscope.

### 2.13 Quantitative reverse transcription polymerase chain reaction (RT-qPCR) and Western blot

MC3T3-E1 cells were cultivated with the materials, and the total RNA of the cells was extracted by the TRIzol method after 21 days. The mRNA profiles of genes associated with osteogenesis (including Alkaline phosphatase (ALP), type Ⅰ Collagen (COL-1), Osteoblastin (OPN) and Runt-related transcription factor 2(RUNX-2) were quantified employing a real-time PCR kit. Transcript standards of all genes were normalised using the housekeeping gene β-actin.

In addition, after co-culture of MC3T3-E1 cells with samples, proteins from the cells were extracted and the expression standards of proteins associated with osteogenesis (including ALP, COL-1, OPN and RUNX-2) in different groups measured by Western blot.

### 2.14 In vivo experiment

#### 2.14.1 Construction of the mouse model and the animal experiment

A total of 12 male Sprague-Dawley rats, aged 8–10 weeks, were randomly assigned to one of six groups, namely control group, Sr-Alg group and Sr-LBP/Alg group, with four rats in each group. Intratracheal inhalation anaesthesia was used, and the right lower limbs of the rats were disinfected with iodine povidone. A small incision was made on the lateral side of the lower and middle femurs of the rats, and the cortex of the femur was exposed layer by layer, and a full-length bone defect of 3 mm in diameter was constructed on the lateral distal femur using an electric drill. Rats in the control group were rinsed with sterile saline and the defects were left empty for suturing after modelling. In the Sr-Alg group, Srcl2 solution and Alg solution were injected directly into the bone defect area of rats and the two liquids were mixed and crosslinked in situ to form a Sr-Alg hydrogel. In the Sr-LBP/Alg group, Srcl_2_ solution and LBP/Alg solution were injected directly into the bone defect area via a dual-channel syringe, and the two liquids were mixed and crosslinked in situ to form a Sr-LBP/Alg hydrogel. Sterile saline was used to rinse and then suture the incision. The rats were sacrificed at 8 weeks after surgery. Miscro-CT, HE staining and immunofluorescence staining were adopted to evaluate repair of the bone defects at 8 weeks.

## 3 Results and discussion

### 3.1 Morphological characteristics of Sr-LBP/Alg hydrogels

**Fig. 2:**
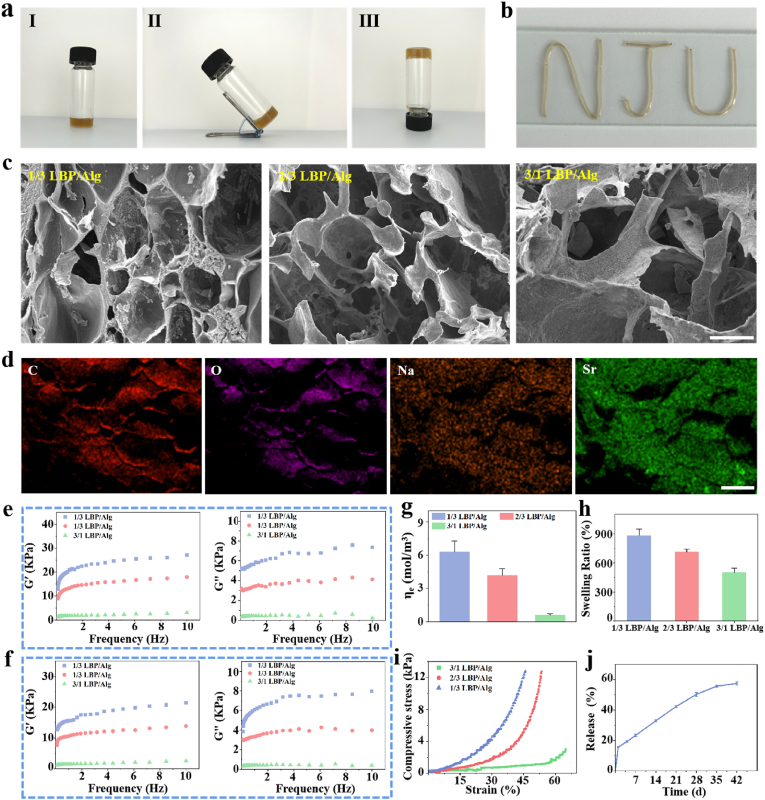
Characterization of Sr-LBP/Alg hydrogel. a) Sr-LBP/Alg hydrogel morphology: I) Sr-LBP/Alg hydrogel after crosslinking, II) Sr-LBP/Alg hydrogel in oblique, and III) Sr-LBP/Alg hydrogel in inverted without changing morphology. b) Injectability of the hydrogel. c) SEM images of LBP/Alg hydrogels with different ratios. Scale bars, 50 μm. d) EDS for Sr-LBP/Alg hydrogel witht ratio of 1/3. Scale bars, 200 μm. e) G′ and G″of Sr-LBP/Alg hydrogels under 25 °C. f) G′ and G″of Sr-LBP/Alg hydrogels under 37 °C. g) The crosslinking density of hydrogels. h) Swelling ratio of LBP/Alg hydrogels with different ratios. i) Compressive stress-strain curves of Sr-LBP/Alg hydrogel at different polymer concentrations. j) LBP released from the composite hydrogel in PBS under 37 °C.

We observed the morphology of the hydrogel at different angles (Fig. 2a) and verified the injectability of the hydrogel (Fig. 2b). The impact of varying LBP/Alg ratios on the hydrogel structure was investigated through the use of scanning electron microscopy (SEM) (Fig. 2c). It was observed that the porosity exhibited a gradual decline as the proportion of LBP occupancy increased. The LBP/Alg ratio of 1/3 displayed a more pronounced porous structure. It is postulated that this phenomenon may be attributed to the tendency of LBP molecules in the hydrogel to aggregate as the proportion of LBP increases (Fig. S1). The porosity of hydrogels plays an important role for nutrient exchange [30]. The EDS analysis is performed as described in Fig. 2d. C, O, Sr and Na were detected in the composite hydrogels, which is further evidence that Sr elements were successfully incorporated into Alg hydrogels.

### 3.2 Evaluation of the mechanical properties and physical network structure

The G′ and G″ of the hydrogel were determined from 0.1 to 10 Hz [31]. Fig. 2e-f illustrates the correlation between G′ and G″ and the LBP content. The G′ and G″ of the LBP-coated hydrogels with varying proportions of LBP exhibited distinct values within the analysed frequency ranges of 25 °C and 37 °C. Additionally, it was observed that all G′ values were greater than G″ at both temperatures. It is proposed that the composite hydrogels exhibit robust mechanical stability. The G′ and G″ demonstrated variation with frequency change, which was ascribed to the reorganization and relocation of the LBP and/or Alg polymer chains within the hydrogels. As the proportion of LBP increases, LBP may undergo micrometre-scale self-aggregation in the composite hydrogel. Conversely, a reduction in the proportion of Alg results in a decrease in the number of cross-linking levels between the carboxyl groups of Alg and Sr^2+^. Furthermore, the G′ and G″ exhibited a temperature-dependent behaviour. The G′ and G″ of all composite hydrogels were reduced at 37 °C in comparison to those at 25 °C. Consequently, We can change the proportion of LBP to adjust the mechanical properties of the hydrogel.

In accordance with the RET theory, the alteration in pore size of alginate composite hydrogels at varying LBP/Alg ratios was determined through the utilisation of the storage modulus of swollen hydrogels (Fig. S2). The meaning of pore size was the interval between the effective cross-linking sites of the hydrogel. The mesh size of the Sr-LBP/Alg hydrogels exhibited a significant correlation with temperature, which may be attributed to the increase in temperature, leading to enhanced fluidity of the polymers, thus reducing physical entanglement. Furthermore, the mesh size of the hydrogels exhibited a notable increase with an elevated percentage of LBP. It was postulated that the presence of residual groups in the composite hydrogel resulted in a reduction in the density of cross-linking points (Fig. 2g), thereby leading to an expansion in the effective mesh size [32].

### 3.3 Swelling behaviour

The ability of hydrogel materials to absorb and retain fluids is a significant determinant of their potential utility in biomedical applications. For instance, it serves as a crucial metric for gauging the accessible surface area of hydrogels [33]. The water absorption ability of hydrogels is effected by the ingredients of the gel and the density of the crosslinks. The swelling behaviour of Sr-LBP/Alg hydrogels was evaluated at 37 °C (Fig. 2h). The highest degree of swelling was observed for Sr-LBP/Alg hydrogels with an LBP/Alg ratio of 1/3. The findings indicated that the degree of swelling of the Sr-LBP/Alg hydrogel exhibited a notable decline with the augmentation of the LBP content. The decrease in swelling ratio of sodium alginate-LBP hydrogels was mainly attributed to the synergistic effect of hydrophilic group properties and network structure: the carboxylate of sodium alginate drove swelling through Donnan osmolality, while the nonionic hydroxyl group of LBP enhanced the hydrophilicity of the surfaces (Fig. S3), but its weak hydration ability and network densification caused by chain entanglement together weakened the swelling driving force. In addition, polysaccharide degradation may change the network topology through β-glycosidic bond breakage, creating a swelling inhibition effect.

### 3.4 Pressure strain

We evaluated the compressive strength of hydrogels. The compressive strength of the hydrogels increased as the LBP/Alg ratio decreased from 3/1 to 1/3 (Fig. 2i). The results indicate that the mechanical properties of hydrogels can be adjusted by changing the ratio of polymer components. The mechanical requirements of bone tissue engineering scaffolds are complex, they must provide adequate compressive, tensile and fatigue resistance for load bearing. Typically, scaffolds composed of natural polymers exhibit inadequate mechanical properties for segmental bone regeneration. Cancellous bone typically has a modulus of 10–3000 MPa, and LBP/SA hydrogels have a lower modulus of elasticity than cancellous bone. However, they can be used as load-bearing scaffolds in conjunction with mechanical fixation when bone grafting and fixation are required [34,35]. LBP/SA hydrogel scaffolds can also be used as grafts for rapid filling of bone defects, overcoming the mechanical limitations inherent in bone tissue engineering applications. The mechanical strength of hydrogel scaffolds also plays an important role in cell proliferation and differentiation, and it has been reported in the literature that for hydrogel scaffolds, the preferred modulus for osteogenic differentiation of MSCs is in the range of 11–30 kPa [36]. The elastic modulus of the LBP/SA hydrogel is in the range of 12–20 kPa, and its good mechanical properties are more in line with the requirements of the microenvironment of bone regeneration, thereby achieving a synergistic effect of mechanical adaptation and synergistic effect of osteoinduction.

### 3.5 Release of the LBP and hydrogel degradation experiment

An examination of the release of LBP from the hydrogel over a period of six weeks revealed a sustained release of LBP, with a maximum of 50 % being achieved within the first four weeks (Fig. 2j). This was followed by a gradual decline in the release rate. The results suggest that LBP can be released in a gradual manner within the composite hydrogel system, potentially exerting a prolonged effect on the fracture localization. Further in vivo release assays of LBP similarly demonstrated the slow release properties of the hydrogel system (Fig. S4). We also conducted degradation experiments of the hydrogel, and the degradation curves (Fig. S5) showed that the hydrogel gradually degraded with the extension of time, which shows that the hydrogel system releases the active ingredients to promote bone repair at the same time, and the volume of the hydrogel itself will not hinder the bone repair.

### 3.6 Osteoblast proliferation

**Fig. 3:**
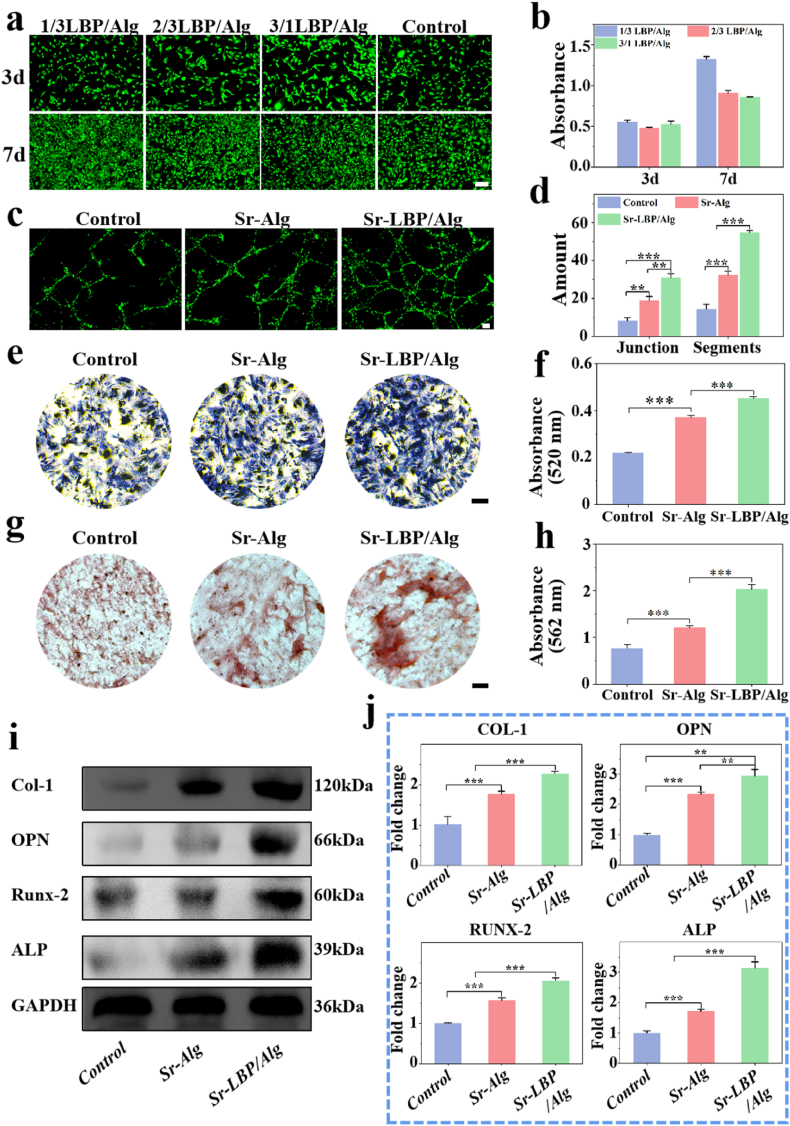
Hydrogel biocompatibility, angiogenesis and osteogenesis in vitro. a) Live/dead staining of MC3T3-E1 cocultured with Sr-LBP/Alg hydrogel for three and seven days in vitro. Scale bars, 200 μm. b) Osteoblast proliferation was determined by CCK8 assay. c) HUVECs tube formation on Matrigel. Scale bars, 200 μm. d) Quantification of endothelial cell tube formation. e) ALP staining of MC3T3-E1 cultured with Sr-LBP/Alg hydrogel for 14 days. Scale bars, 200 μm. f) Quantitative analysis of ALP. g) ARS staining of MC3T3-E1 cultured with Sr-LBP/Alg hydrogel for 14 days. Scale bars, 200 μm. h) Quantitative analysis of ARS. i) Western blot results for osteogenesist-related proteins (Col-1, OPN, RUNX-2 and ALP). j) Quantitative RT-PCR detection of mRNA expression of osteogenesist-related proteins (Col-1, OPN, RUNX-2 and ALP). n = 3, ∗p < 0.05, ∗∗p < 0.01, and ∗∗∗p < 0.001.

Because MC3T3-E11 cells are critical in the repair of bone defects, we examined the activity of MC3T3-E11 cells encapsulated in hydrogels. In this study, Sr-LBP/Alg hydrogel wrapping of the cells was used in different proportions. Both live/dead assay and CCK 8 results showed that hydrogel-coated osteoblasts with a LBP/Alg ratio had the highest proliferation capacity among the three groups after 3 and 7 days of culture(Fig. 3a and b), with positive biocompatibility. The above results are considered related to the elastic modulus and grid size of different proportional hydrogels [37]. So the hydrogels with an LBP/Alg ratio of 1/3 were employed in the following experiments.

### 3.7 Osteogenic and angiogenic differentiation

Osteogenesis and vascularization play a significant role in the healing of bone defects [38,39]. To assess the bone-enhancing and angiogenic effects of hydrogel materials, we employed ALP staining, alizarin red staining, and tube-forming experiments. The results of the angiogenesis experiments (Fig. 3c and d) demonstrated that the Sr-Alg hydrogel exhibited pro-angiogenic effects, with the Sr-LBP/Alg group displaying more pronounced outcomes. ALP staining (Fig. 3e and f), and alizarin red staining (Fig. 3g and h) demonstrated that Sr-Alg hydrogel had favorable bone-enhancing effects, with the results in the Sr-LBP/Alg group exhibiting greater efficacy. The results were deemed to be attributable to the presence of a substantial concentration of Sr ions in the Sr-Alg hydrogel, which exhibited osteogenic and angiogenic effects [40]. With the incorporation of LBP, the osteoinductive and angiogenic effects of the material were further enhanced. Further quantitative analysis also showed that Sr-LBP/Alg promoted the formation of mineralized nodules and blood vessels.

The qRT-PCR results demonstrated that the expression standards of the osteoblast differentiation biomarker genes ALP, OPN, and COL-1 (Fig. 3j) were markedly elevated in the Sr-Alg and Sr-LBP/Alg groups relative to the control group, with a more pronounced increase observed in the Sr-LBP/Alg group. The ALP and COL-1 genes have a pivotal effect on bone mineralization, and the enhancement of mineralization facilitates the formation of bone [41]. The transcription factor RUNX-2 plays a pivotal function in the differentiation of osteoblasts and the spectrum-specific regulation at the transcriptional level [42]. Additionally, the present study revealed a notable elevation in RUNX-2 expression levels in the Sr-Alg and Sr-LBP/Alg groups. Western blot analysis revealed that Sr-LBP/Alg hydrogel intervention resulted in a meaningful raise in the expression standards of osteogenesis-related proteins, including ALP, OPN, COL-1, and RUNX-2 (Fig. 3i). Thereforce, the above results indicate that hydrogels have positive osteogenic effects in vitro.

### 3.8 In vivo experiment

**Fig. 4:**
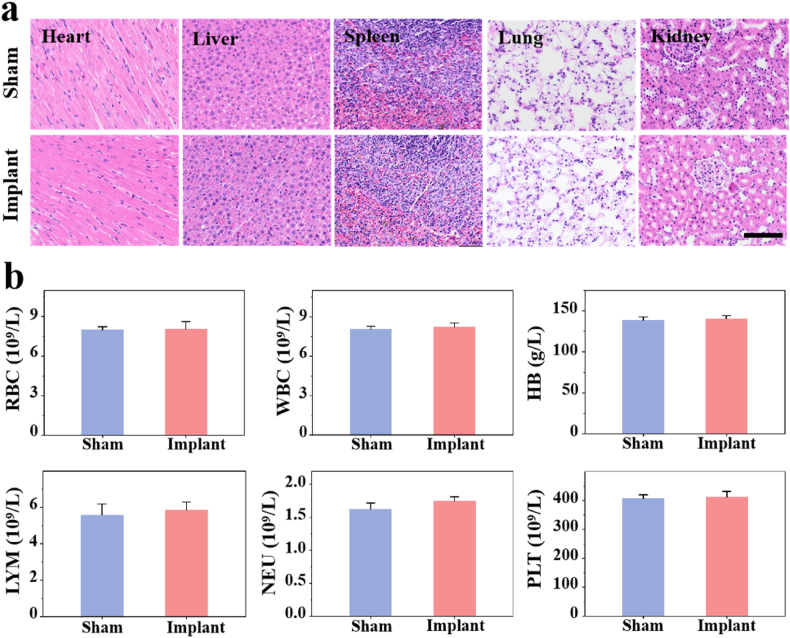
Material compatibility experiments in vivo.a) Visceral HE staining of the sham and implant group rats. Scale bars, 100 μm. b) Results of routine blood tests in both groups.

The Sr-LBP/Alg hydrogel has been demonstrated to exhibit favorable cytobiocompatibility and in vitro osteoinduction properties. In this study, the osteogenic effect of the hydrogel system was further assessed by rat femoral defect model. To ascertain the toxicity of the hydrogel in animals, histologic staining of the major organs of the rats was conducted, and the findings indicated that the hearts, livers, spleens, lungs, and kidneys of the SD rats that had undergone hydrogel implantation did not demonstrate any notable inflammation or damage (Fig. 4a). Furthermore, peripheral blood was drawn from SD rats for routine blood tests, and the results demonstrated no evident abnormalities (Fig. 4b). In conclusion, these findings indicate that the hydrogel exhibits positive biocompatibility.

**Fig. 5:**
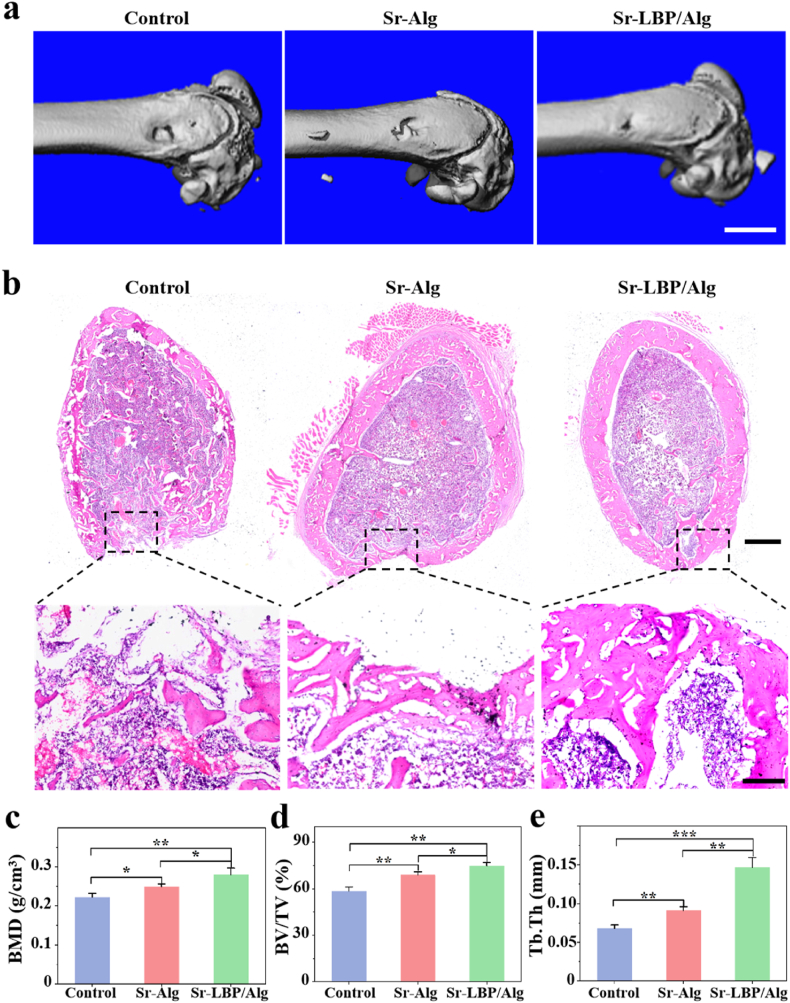
Micro-CT and HE staining evaluated the effect of injectable composite microspheres on bone regeneration at 8 weeks. a) Micro-CT images. Scale bars, 5 mm. b) HE staining. Scale bars, 1 mm and 200um. c) Quantitative analysis of BMD, BV/TV ratio and Tb.Th. n = 3, ∗p < 0.05, ∗∗p < 0.01, and ∗∗∗p < 0.001.

Next, we tested whether the Sr-LBP/Alg hydrogel system could facilitate enhanced bone healing in rats with critical-size femoral defects. After 8 weeks of intervention, regenerated bone was visualised and quantified using micro CT scanning. The newly formed tissue connected with the surrounding tissue and the bone defect area was significantly filled with repair 8 weeks after injection, compared to the control rats with limited filling of the femoral bone defect with localized bone repair (Fig. 5a). The osteogenic capabilities of the hydrogel were additionally evaluated through HE staining (Fig. 5b), which showed more new bone formation in the Sr-LBP/Alg hydrogel hydrogel group at week 8. The CT scan data were subjected to morphometric analysis (Fig. 5c–e), which yielded further confirmation that the parameters were significantly higher in the Sr-Alg and Sr-LBP/Alg hydrogel groups. Moreover, these differences were more pronounced in the Sr-LBP/Alg group, including the relative tissue volume (BV/TV), bone mineral density (BMD), and the thickness of the trabeculae of bone (Tb.Th).

**Fig. 6:**
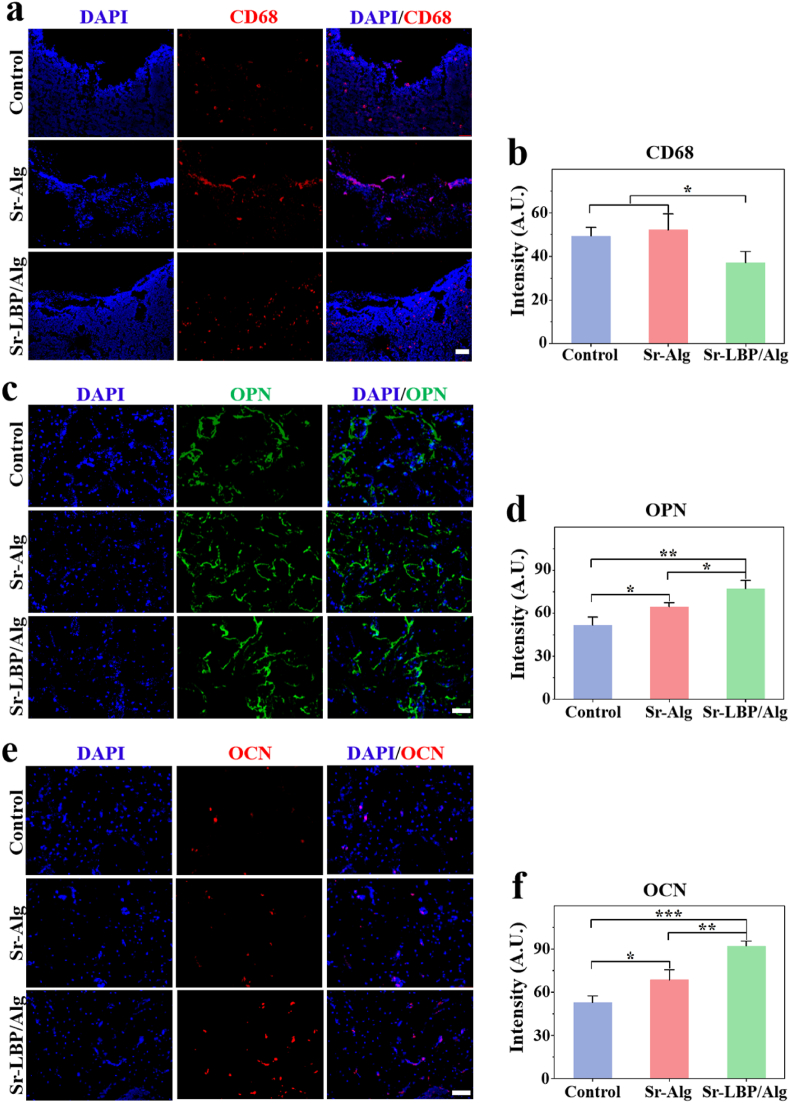
Histological immunofluorescence analysis in vivo. a,b) CD68 immunofluorescence staining and quantification of fluorescence intensity. c,d) OPN immunofluorescence staining and quantification of fluorescence intensity. e,f) OCN immunofluorescence staining and quantification of fluorescence intensity. Scale bars, 100 μm. n = 3, ∗p < 0.05, ∗∗p < 0.01, and ∗∗∗p < 0.001.

To further detect the presence of inflammation and bone regeneration in the bone defect area, we performed immunofluorescence staining. The results demonstrated that the expression of CD68 in the Sr-LBP/Alg group was markedly diminished in comparison to the other groups at postoperative weeks 4 and 8 (Fig. 6a and b). This indicated that the Sr-LBP/Alg group exhibited the least degree of local inflammation among the three groups, suggesting that LBP has the potential to improve the local inflammatory environment of fractures. Furthermore, our findings revealed that the Sr-LBP/Alg group exhibited the highest immunofluorescence intensity for osteogenesis-related protein detection. The quantitative analysis demonstrated that the expression of osteoblast markers OPN (Fig. 6c and d) and OPN (Fig. 6e and f) in the Sr-LBP/Alg group was markedly elevated in comparison to the other groups at the eighth week post-surgery. This indicated the osteogenic promotion effect of Sr-LBP/Alg hydrogel, and the addition of LBP could further enhance the osteogenic effect of Sr-Alg hydrogel. In summary, it was shown that Sr-LBP/Alg hydrogel significantly promoted osteogenesis both in vitro and vivo.

This research also has some limitations. Some studies have proved that LBP has the effect of strengthening muscles and bones, and in this study, the design of LBP concentration was not refined enough and could not propose a more precise proportion of LBP intervention concentration. Second, this study could not conduct in-depth research on the mechanism level. Therefore, our future research will focus on exploring the optimal concentration of LBP and the specific pathway of LBP to promote bone regeneration.

## 4 Conclusion

In this study, we successfully developed an injectable in situ rapidly crosslinked Sr-LBP/Alg hydrogel system: we have successfully combined the trace element Sr and traditional Chinese medicine polysaccharide in Alg hydrogel. Both in vitro and vivo experiments showed that the addition of LBP enhanced the osteoinductive effect of the hydrogel. The composite hydrogel also promoted angiogenesis and bone mineralization in the area of bone defects, which are essential for bone regeneration. In addition, the bioactive hydrogel composite scaffold has positive biocompatibility in vivo. In conclution, Sr-LBP/Alg hydrogel is a promising material for bone regeneration.

## CRediT authorship contribution statement

**Kai Xie:** Writing – original draft, Methodology. **Zhengwei Liu:** Software, Methodology. **Yansong Wang:** Resources, Investigation. **Lei Zhu:** Methodology, Investigation. **Jiazhao Yang:** Data curation, Conceptualization. **Yongxiang Wang:** Visualization, Validation, Supervision. **Mingming Zhang:** Writing – review & editing, Project administration, Conceptualization.

## Availability of data and materials

The data generated in this work is available from the corresponding author on reasonable request.

## Ethics approval and consent to participate

Relevant studies have been complied with the ARRIVE guidelines and the National Research Council's Guide for the Care and Use of Laboratory Animals, and conducted according to the standard guidelines approved by the Animal Experimentation Ethics Committee of Nanjing University Medical School (2023AE01019).

## Consent for publication

All authors agree to publication.

## Funding

This work was supported by 10.13039/501100002949Jiangsu Province "333 project" key industry talents (BRA202201) and Yangzhou science and technology plan (YZ2022070, YZ2023266).

## Declaration of competing interest

The authors declare that they have no known competing financial interests or personal relationships that could have appeared to influence the work reported in this paper.

## Data Availability

Data will be made available on request.
