# Supplementary material for: Injectable functionalised Lycium barbarum polysaccharide/alginate hydrogel particles for the treatment of bone defects
Source: Mater Today Bio. 2025 May 28;32:101919. doi: 10.1016/j.mtbio.2025.101919 (PMC12163170; doi:10.1016/j.mtbio.2025.101919)
Supplement: Multimedia component 1 [file mmc1.docx]

**Supplementary Information**

**Injectable functionalised Lycium barbarum polysaccharide/Alginate hydrogel particles for the treatment of bone defects**

Kai Xie^1,†^, Zhengwei Liu^1,†^, Yansong Wang^2,†^, Lei Zhu^1^, Jiazhao Yang^3,*^, Yongxiang Wang^1,*^, Mingming Zhang^4,*^


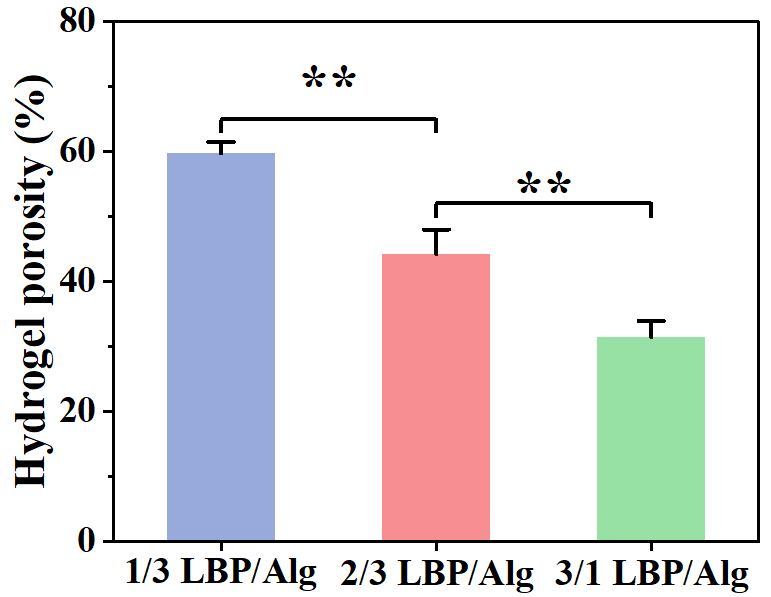


**Figure S1.** Quantitative analysis of porosity of hydrogels of different compositions by Imajej.


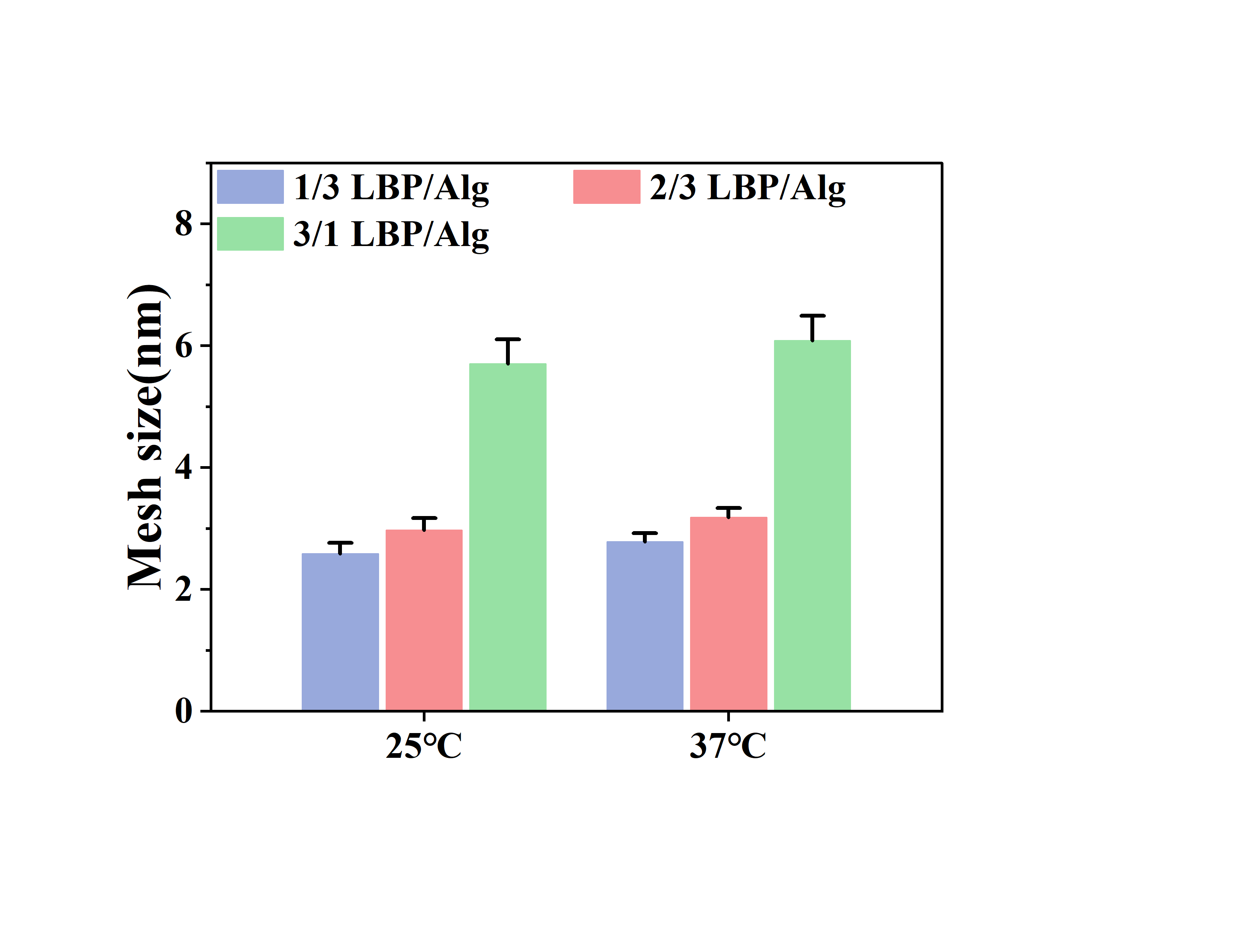


**Figure S2.** The Mesh size of hydrogels in different proportions.


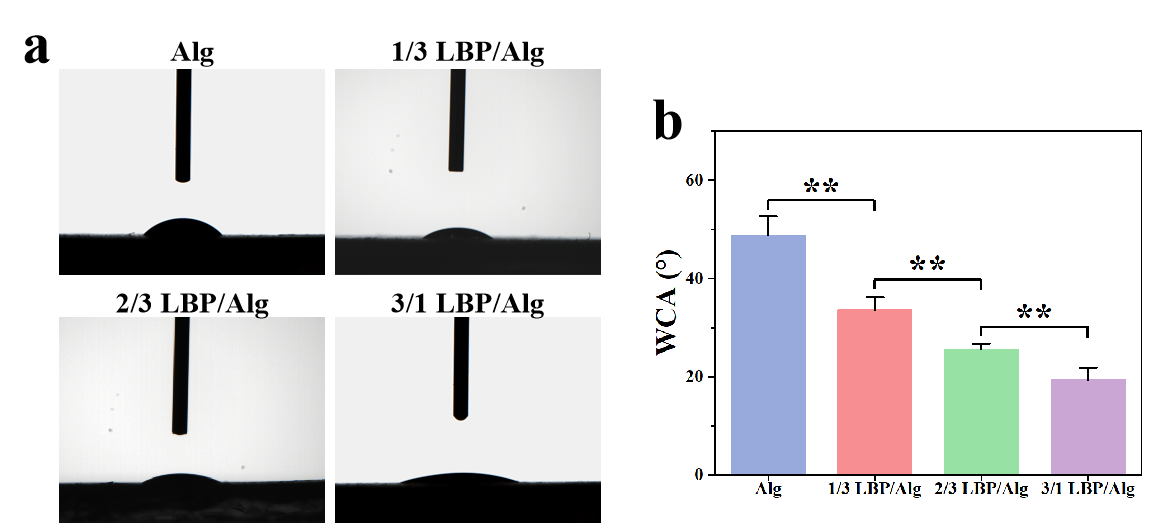


**Figure S3.** Hydrophilicity test results of hydrogels. a) Photographs of water contact angle (WCA) measurements of different hydrogel materials. b) Quantitative analysis of water contact angle of different hydrogel materials.


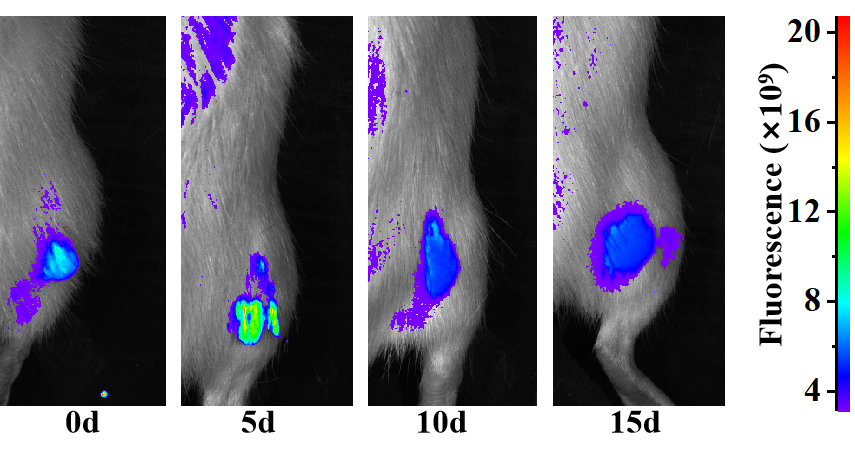


**Figure S4.** Results of animal imaging techniques to detect LBP release in vivo.


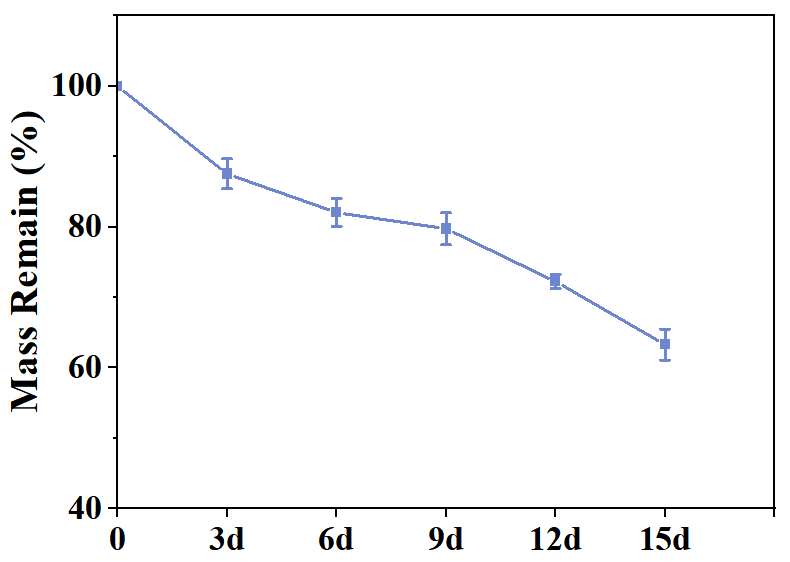


**Figure S5.** Degradation curve of hydrogel.

**Table S1.** qPCR primers

| ***Gene*** | **Sequences(5′–3′)** |
| --- | --- |
| *ALP* | F: CTGGACTTGGTGGTCACAGC |
|  | R: CGGGACGAGGAAACACTCTC |
| *OPN* | F: ATCCAAGGATGCCAACGACTC |
|  | R:AGCCATTGACCACCAAGAAGC |
| *RUNX-2* | F: CGCCTCACAAACAACCACAG |
|  | R: TGCTTGCAGCCTTAAATATTCCTG |
| *COL-1* | F: ACGCCATCAAGGTCTACTGC |
|  | R: ACTCGAACGGGAATCCATCG |
| *GAPDH* | F: AAGAGGGATGCTGCCCTTAC |
|  | R: CGGGACGAGGAAACACTCTC |
